# In silico optical modulation of spiral wave trajectories in cardiac tissue

**DOI:** 10.1007/s00424-023-02889-7

**Published:** 2023-12-14

**Authors:** Sayedeh Hussaini, Rupamanjari Majumder, Valentin Krinski, Stefan Luther

**Affiliations:** 1 Institute of Pharmacology and Toxicology, University Medical Center Göttingen, Robert-Koch-Straße 40, 37075 Göttingen, Niedersachsen Germany; 2 Research Group Biomedical Physics, Max Planck Institute for Dynamics and Self-Organization, Am Fassberg, 37077 Göttingen, Niedersachsen Germany

**Keywords:** Sub-threshold stimulation, Optogenetics, Cardiac excitability, Feedback pacing

## Abstract

Life-threatening cardiac arrhythmias such as ventricular tachycardia and fibrillation are common precursors to sudden cardiac death. They are associated with the occurrence of abnormal electrical spiral waves in the heart that rotate at a high frequency. In severe cases, arrhythmias are combated with a clinical method called defibrillation, which involves administering a single global high-voltage shock to the heart to reset all its activity and restore sinus rhythm. Despite its high efficiency in controlling arrhythmias, defibrillation is associated with several negative side effects that render the method suboptimal. The best approach to optimize this therapeutic technique is to deepen our understanding of the dynamics of spiral waves. Here, we use computational cardiac optogenetics to study and control the dynamics of a single spiral wave in a two-dimensional, electrophysiologically detailed, light-sensitive model of a mouse ventricle. First, we illuminate the domain globally by applying a sequence of periodic optical pulses with different frequencies in the sub-threshold regime where no excitation wave is induced. In doing so, we obtain epicycloidal, hypocycloidal, and resonant drift trajectories of the spiral wave core. Then, to effectively control the wave dynamics, we use a method called resonant feedback pacing. In this approach, each global optical pulse is applied when the measuring electrode positioned on the domain registers a predefined value of the membrane voltage. This enables us to steer the spiral wave in a desired direction determined by the position of the electrode. Our study thus provides valuable mechanistic insights into the success or failure of global optical stimulation in executing efficient arrhythmia control.

**Supplementary Information:**

The online version contains supplementary material available at 10.1007/s00424-023-02889-7.

## Introduction

Anomalies in the generation and conduction of electrical signals in the heart lead to instabilities, which often result in the formation and sustenance of spiral excitation waves. These rotating waves are associated with life-threatening cardiac rhythm disorders, such as ventricular tachycardia (VT) and ventricular fibrillation (VF) [11, 12, 21]. In the clinic, a method called anti-tachycardia pacing (ATP) is frequently used to terminate VT. In this technique, a sequence of high-frequency electrical pulses is applied locally to the cardiac tissue. Each induced electrical pulse generates an excitation wave that propagates through the cardiac tissue toward the organizing center or core of the spiral wave. This eventually leads to the penetration of the excitation waves into the spiral core. Each penetrating wave pushes, and thereby displaces, the tip of the spiral wave, causing it to drift. The drift may be towards a non-excitable boundary or another spiral wave, with which it collides and annihilates [17, 29]. However, the ATP method fails to control high-frequency VT (> 188–250 bpm) [32, 35]. Such high-frequency VTs and VF are controlled by applying a single high-voltage electric shock to the heart. This causes global excitation of the cardiac tissue, resetting all electrical activity and allowing the natural pacemaker to re-establish a regular rhythm. However, despite the high success rate, this method is associated with numerous harmful side effects such as severe pain, trauma, anxiety, and depression [16, 27, 34]. Therefore alternative less harmful methods with comparable efficiency are being intensively researched [2, 14, 22, 24]. Further advances in the clinical application of these emerging techniques require a deeper understanding of the dynamics of the spiral waves rotating in cardiac tissue during arrhythmia. Such detailed investigations require experimental tools/technologies that make it possible to verify and validate the results obtained with the help of mathematical models. In this respect, optogenetics has shown great potential in cardiac research, providing not only an effective means to control life-threatening arrhythmias with low energy expenditure in small animal hearts [3, 9] but also, a resourceful research tool to study spiral wave dynamics and the diverse mechanisms underlying successful defibrillation at sub-/suprathreshold light intensities [4, 20, 26].

Conceptually, optogenetic stimulation differs from electrical stimulation. During optical stimulation, the membrane voltage is affected indirectly, through the activation of light-responsive membrane proteins. However, for electrical stimulation, the membrane voltage is directly elevated by the externally applied electrical current. With suprathreshold local stimulation, in both the electrical and optical cases, the tissue depolarises similarly to generate a new excitation wave. Thus the overall effect of the stimulation on cardiac tissue is similar in the two cases, with the potential to terminate only monomorphic arrhythmias. Global (field) stimulation, on the other hand, is a different story. Assuming the homogeneity of expression of the light-gated ion channel, and the characteristic property of light that its amplitude decays exponentially with depth inside cardiac tissue, global optical stimulation mainly tends to control arrhythmias through the closure of the excitable gap. However, global electrical stimulation results in indirect depolarization of the tissue, where heterogeneities become new sources for the generation of excitation waves [24, 29]. This leads to a global inhomogeneous depolarization of the tissue. In both optical and electrical cases, polymorphic arrhythmias can be successfully terminated.

In a previous study [20], we investigated light-based control of a single spiral wave in a two-dimensional (2D) model of optogenetically-modified mouse ventricular tissue, by spatial modulation of the excitability. Our studies revealed the possibility of inducing spiral wave drift, which eventually led to arrhythmia termination. Recently, Li et al. [23] investigated the control of spiral wave dynamics in optogenetically modified 2D cardiac tissue using temporal modulation of excitability. Their study reports a similar drift of the spiral wave at stimulation frequencies close to the frequency of the spiral. Thus, motivated by these findings, here we present a theoretical study of spiral wave dynamics in the presence of global periodic, subthreshold illumination. We show that the spiral frequency changes due to light-induced elevation of the membrane potential of the cells (and change in conduction velocity). This leads to interesting meander patterns in a system that otherwise supports stationary spirals. Thus, through our study, we are able to provide an explanation for why fixed-frequency optical stimulation sometimes fails to effectuate spiral wave termination. Based on the knowledge thus gathered, we explore the applicability of an advanced technique to control single spiral wave-associated VTs in cardiac tissue.

To understand what exactly happens during fixed-frequency optical stimulation, we investigate spiral wave dynamics in a two-dimensional (2D) mouse ventricular model, which is subject to temporal modulation of excitability. We apply a sequence of periodic sub-threshold globally illuminating optical pulses with different frequencies to the simulation domain. We call this method, the method of open-loop periodic pacing. Depending on the optical pacing frequency, we observe different core dynamics, such as complex meander patterns of the spiral wave (epicyclodal, hypocycloidal, etc.), when the latter is not terminated. This leads us to conjecture that fixed-frequency stimulation can cause stationary spiral waves to start meandering (often without eventual termination) if these waves are located far from non-excitable boundaries or the vicinity of other spiral waves.

Based on this theory, we modify the pacing protocol to try a more advanced, control approach, originally proposed by [1, 6, 13, 33]. Thus, we apply a closed-loop pacing method (we call this method *resonant feedback pacing*), to the same system. Here, the membrane voltage is recorded continuously by a measuring electrode from a chosen location within the simulation domain. Every time the recorded voltage supercedes a preset value (− 40 mV), we apply a global optical pulse to the system. This stimulation protocol allows us to steer the spiral wave in a desired direction determined by the position of the measuring electrode. Thus, we present a technique that clearly allows better control of VT than existing conventional techniques.

## Methods

Numerical simulations were performed in a 2D domain of the mouse ventricle. This domain was constructed using the ionically detailed mathematical model of a mouse ventricular cardiomyocyte, proposed by Bondareko et al. [8, 28]. To calculate the temporal evolution of the electrical activity across the membrane, 40 ordinary differential equations were solved using the Runge–Kutta method (4th order) with a temporal resolution of dt = 10^−4^ ms. Here, the temporal evolution of the membrane voltage activity is described as follows:

1 $$\frac{dV}{dt}=-\frac{\sum {I}_{ion} + {I}_{stim}}{{C}_{m}}$$

2 $$\sum {I}_{ion}= {I}_{Ktof}+{I}_{Ktos}+{I}_{Kr}+{I}_{Kur}+{I}_{Kss}+{I}_{K1}+{I}_{Ks}+{I}_{Na}+{I}_{Ca}{+ I}_{NaCa}+{I}_{Ca}+{I}_{Nak}+{I}_{CaCl}+{I}_{Nab}+{I}_{Cab}$$

where V (mV) is the membrane voltage, C_m_ (µF*/*cm^2^) is the membrane capacitance. *I*_ion_ is the net ionic current flowing across the cell membrane, as a sum of 15 different ionic currents: fast Na^+^ current (I_Na_), L-type Ca^2+^ current (*I*_CaL_), Ca^2+^ pump current (*I*_pCa_), rapid recovering transient outward K^+^ current (*I*_Kto,f_), slow recovering transient outward K^+^ current (*I*_Kto,s_), rapid delayed rectifier K^+^ current (*I*_Kr_), ultrarapid activating delayed rectifier K^+^ current (I_Kur_), non-inactivating steady-state voltage-activated K^+^ current (*I*_Kss_), time-independent inwardly rectifying K^+^ current (I_K1_), slow delayed rectifier K^+^ current (I_Ks_), Na^+^/Ca^2+^ exchange current (I_NaCa_), Na^+^/K^+^ pump current (*I*_NaK_), the Ca^2+^-activated Cl^−^ current (*I*_Cl,Ca_), background Ca^2+^ current (*I*_Cab_), background Na^+^ current (*I*_Nab_) and stimulation current (*I*_stim_) which is an external current and can trigger an action potential in a single cell.

To incorporate light-sensitivity to the cardiac cell we coupled a 4-state model of a light-responsive protein Channelrhodopsin-2 (ChR2) [36]. The main equations for the model are described below:

3 $$I_{ChR2}=g_{ChR2}G\left(V\right)\left(O_1+\gamma O_2\right)\left(V-E_{ChR2}\right)$$

4 $${dC}_1/dt=G_rC_2+G_{d1}O_1-k_1C_1$$

5 $${dO}_1/dt=k_1C_1+\left(G_{d1}+e_{12}\right)O1+e_{21}O_2$$

6 $${dC}_2/dt=G_{d2}O_2-\left(k_2+G_r\right)C_2$$

7 $${dO}_2/dt=k_2C_2-\left(G_{d2}+e_{21}\right)O_2+e_{12}O_1$$

8 $$G\left(V\right)=\left[\left(10.6408-14.6408\times exp\left(-V/42.7671\right)\right)\right]/V$$

Here g_ChR2_ is the maximum conductance, O_1_ and O_2_ are the open states of ChR2, *γ* = 0*.*1 is, probability, the ratio of contribution of open states O_2_/O_1_, V is the membrane voltage, and *E*_*ChR*2_ is the reversal potential, which was taken to be 0 mV. G_1_,

G_d1_, G_d2_, e_12_, e_21_, k_1_, and k_2_ are the kinetic parameters corresponding to the transition states of ChR2 states (close: C1, C2 and open: O1, O2). G(V) is the voltage dependent rectification function. The model parameters are described and explained in detail in Ref [36].

9 $$G\left(V\right)=\left(10.6408-14.6408\times \mathrm{exp}\left(-V/42.7671\right)\right)$$

In 2D, we solved a partial differential equation for the membrane voltage (with a spatial resolution of dx = dy = 0*.*025 cm) which is described as follows:

10 $$\frac{dV}{dt}=\nabla .\mathcal{D}\nabla V-\frac{{I}_{ion} + {I}_{ChR2}+ {I}_{stim}}{{C}_{m}}$$

$\mathcal {D}$ is the diffusion coefficient with a value of 0*.*00014 cm^2^*/*ms to produce a conduction velocity of 43.9 cm/s. The domain contained 100 × 100 grid points and an excitation wave propagated through it with a conduction velocity of 43.9 cm/s. This equation was solved using the finite difference method with 5-point stencil.

## Results

**Fig. 1:**
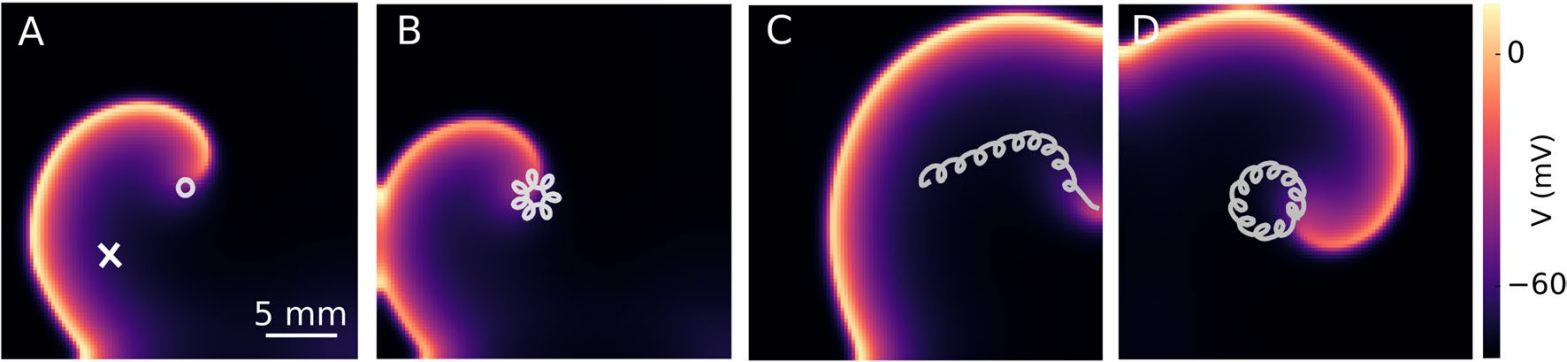
Control of a single spiral wave’s dynamics using global open-loop periodic illumination at sub-threshold regime. **A** A single spiral wave rotating in a homogeneous two-dimensional region in the absence of light. The tip of the spiral wave traces a circular trajectory with a frequency of 15.6 Hz. **B** A spiral wave with a core of hypocycloidal meandering pattern induced by applying global optical pulses with a pacing frequency (*f*_p_) of 15*.*3 Hz. **C** A spiral wave with an induced resonance drift using a global periodic illumination at *f*_p_ = 13*.*33 Hz. **D** A spiral wave with a core of epicycloidal meandering pattern induced by global optical pulses with *f*_p _= 12*.*5 Hz. The cross marker in **A** shows an electrical measuring point

Figure 1A shows a single spiral wave in a 2D domain of mouse ventricular tissue which rotates with a frequency (*f*_s_) of 15*.*6 Hz. After a period of rotation (66 ms), the tip of this wave forms a core with a circular shape, as shown in Fig. 1A. To control the dynamics of this single spiral wave, we modulated the excitability of the domain using open-loop periodic pacing. In this method, we apply a sequence of global optical pulses of sub-threshold intensity (20 µW*/*mm^2^), 33 ms pulse length (half a period of the spiral wave rotation), and three different pacing frequencies (*f*_p_): I) *f*_p_ = *f*_s_ = 15*.*6 Hz, II) *f*_p_ = 13*.*33 Hz, and III) *f*_p_ = 12*.*5 Hz. Figure 1B, C show the spiral wave with different core dynamics: (B) hypocycloidal, induced by the *f*_p_ of 15*.*6 Hz, **C** resonant drift, induced by the *f*_p_ of 13*.*33 Hz, and **D** epicycloidal, induced by the *f*_p_ of 12*.*5 Hz.

**Fig. 2:**
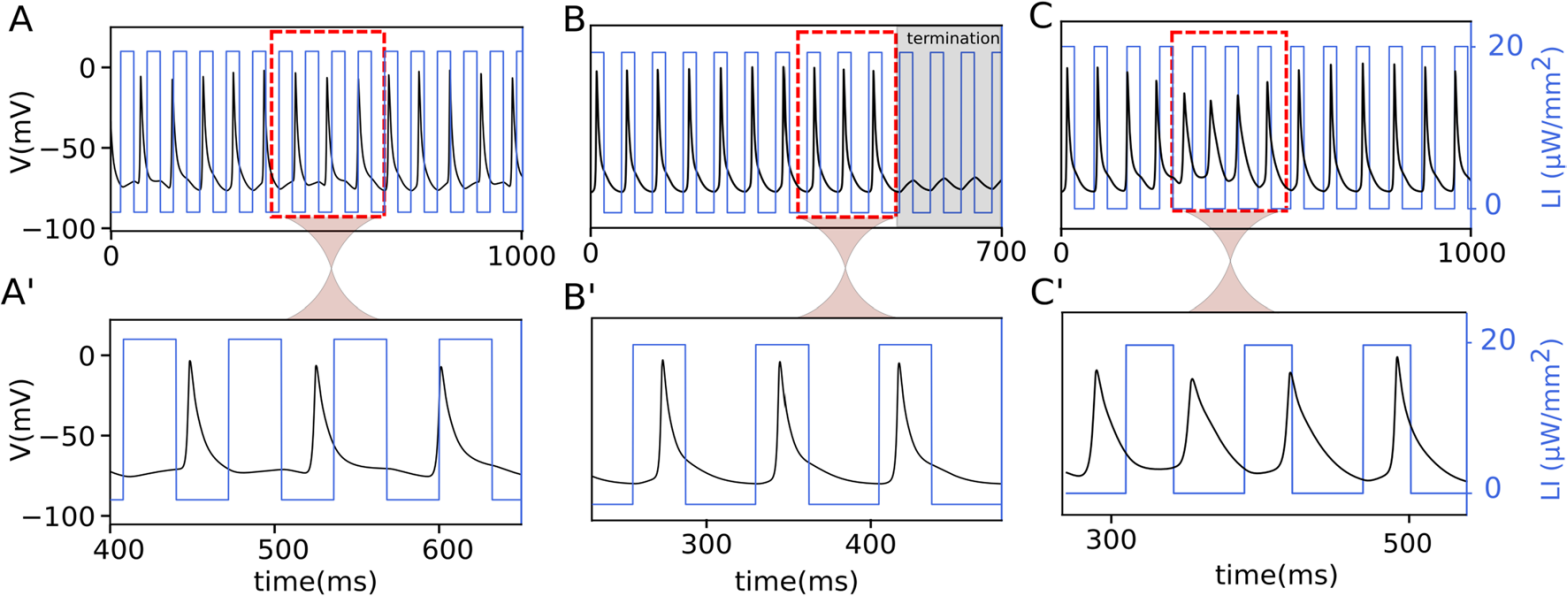
Electrical activity (black trace) of a spiral wave during global periodic illumination (blue lines). **A**–**C** Voltage–time series of a spiral wave, when the domain is illuminated globally with a pacing frequency of 15.3 Hz in (**A**), 13.33 Hz in (**B**), and 12.5 Hz in (**C**). The gray box in (**B**) highlights the termination of the spiral wave. **A**–**C** A closer look at a segment of the voltage–time series in (**A**–**C**), marked with a dashed red box. In all cases, the optical pulse has an amplitude of 20 µW*/*mm^2^

To understand how illumination affects the dynamics of the spiral wave, we examined the electrical activity at a representative point (indicated with a cross marker in Fig. 1A) within the simulation domain. Figure 2A–C (solid black line) illustrates the temporal electrical activity recorded from this point over a period of 1000 ms. The applied optical pulses are shown using overlaid solid blue lines. In all three cases, the voltage–time series shows that the frequency *f*_s_ decreased during periodic illumination. Figure 2A–C shows that the illuminated *f*_s_ decreased to 13 Hz, 13.33 Hz, and 14 Hz, respectively, compared to the unilluminated case with an *f*_s_ value of 15.6 Hz (a comparison between the voltage time series of the spiral wave before and during illumination, shown in Supplementary Figure S1). Figure 2A–C shows an expanded view of the part of the time series marked out with a dashed red box in the corresponding panel above. Figure 2A shows that the number of optical pulses is greater than the number of action potentials, which means that *f*_p_ > *f*_s_, thus the spiral wave is paced overdrive. This leads to hypocycloidal core dynamics of the spiral wave with a meander pattern characterized by outward pointing petals as shown in Fig. 1B. In contrast, Fig. 2B shows an equal number of optical pulses as the number of action potentials (*f*_p_ = *f*_s_). Thus the spiral wave is paced resonantly. This leads to a resonant drift of the spiral wave, causing it to ultimately causes the wave to collide with the unexcitable boundary after 10 optical stimuli and terminate (see Fig. 1C and gray box in Fig. 2B). Figure 2C shows the case where the number of optical pulses is less than the number of action potentials (*f*_p_ < *f*_s_). Thus the spiral wave here is paced underdrive. This leads to epicycloidal core dynamics of the spiral wave, with a meander pattern characterized by inward-pointing petals as shown in Fig. 1D.

**Fig. 3:**
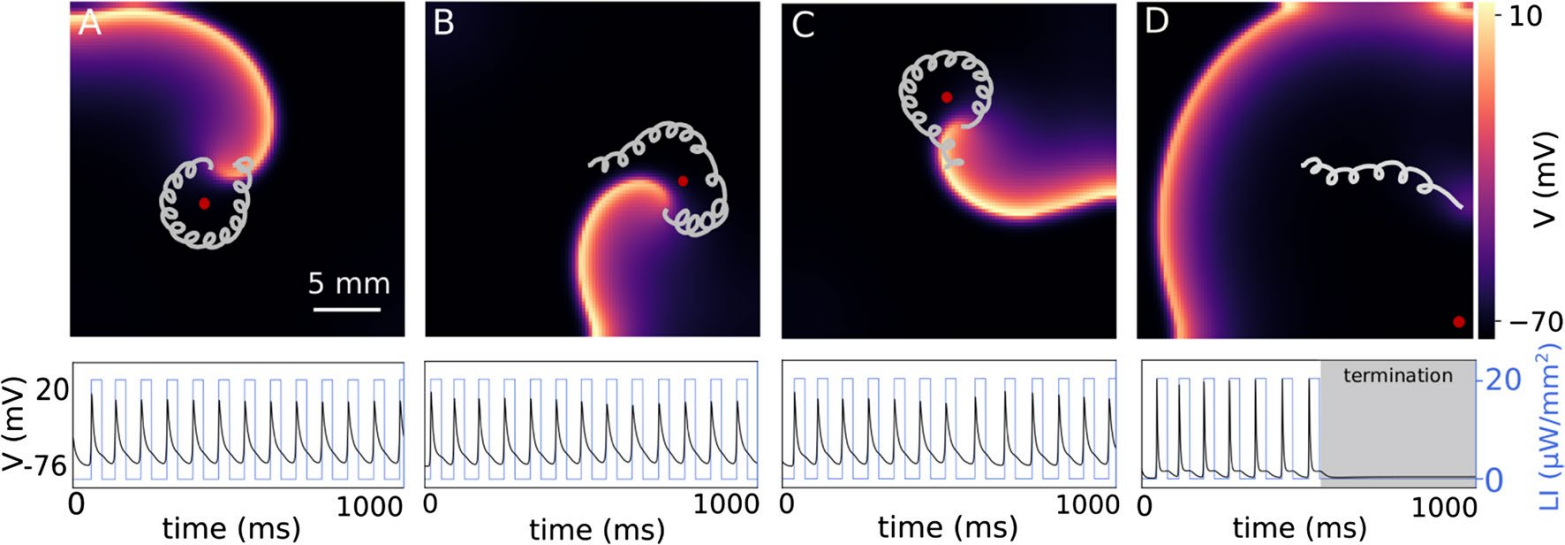
Spiral wave control using closed-loop global repetitive sub-threshold illumination. **A**–**D** The upper panel illustrates the core dynamics of a single spiral wave in the presence of resonant feedback pacing when the measuring electrode, indicated by a red dot, is located at different coordination. The corresponding voltage series (black trace) recorded from the location of the measuring electrode is shown together with the overlaid optical signal (blue line) in the panel below. In all cases, the light intensity is 20 µW*/*mm^2^

Temporal modulation of cardiac excitability leads to termination of the spiral wave when *f*_p_ is in resonance with *f*_s_. This leads to resonant drift of the spiral wave and its termination upon collides with a nearby non-excitable boundary. Furthermore, we showed that illumination slows down the dynamics of the spiral wave as its frequency decreases. This deceleration depends strongly on the light intensity and the pacing frequency. Therefore, determining a pacing frequency that causes resonant drift of a spiral wave at a given light intensity is a major challenge. To overcome this hurdle, we employed a closed-loop pacing approach named resonant feedback pacing. In this method, we placed a measuring electrode in the domain to record the electrical activity. We defined a critical voltage (*Vc*) with a value of − 40 mV. Whenever the wavefront crossed this measuring point so that the measured voltage surpassed *Vc*, global subthreshold illumination was applied to the domain. This resulted in modulating the excitability of the domain with a sequence of repetitive global optical pulses at a desired sub-threshold light intensity. This method naturally stops applying optical stimulation once the recording electrode is unable to detect the electrical activity of an excitation wave within the domain, i.e., when the spiral wave is terminated. To control and steer the single spiral wave in our 2D domain, we placed the measuring electrode at different positions and let the system evolve for 1000 ms. Figure 3A–D shows the dynamics of the spiral wave when the sensing electrode is located at coordinates (1 cm, 1 cm), (1.875 cm, 1.25 cm), (1.25 cm, 1.875 cm), and (2.4 cm, 0.1 cm), respectively. The electrical activity measured by the sensing electrode is shown below for each case. The optical pulses, with the amplitude of 20 µW*/*mm^2^ and width of 33 ms, are applied when the voltage exceeds *Vc* =  − 40 mV during the depolarization phase of the action potential. In Fig. 3A–C, we observed that the tip of the spiral wave forms a meandering pattern, with petals pointing inwards, around the measuring electrode. This is due to the slight displacement of the wavefront with respect to the electrode after each rotation. Therefore, placing the measuring electrode in a corner of the domain causes the spiral wave to drift towards one of the unexcitable borders as it is on its way to form a circular, meandering path around the electrode. This leads to the collision of the wave with the boundary and effectuates its termination (see Fig. 3D). The electrical activity shown below illustrates that the membrane voltage goes to the quiescent state after seven optical pulses. This means that there is no excitation wave propagating in the domain, i.e., the spiral wave has been terminated.

## Discussion

Spiral waves rotating in excitable media are examples of self-organized phenomena observed in many chemical and biological systems such as the Belousov-Zhabotinsky (BZ) reaction and the heart [19, 30]. Basic investigations of spiral wave dynamics, such as meander and resonant drift, have been carried out in numerous theoretical and experimental studies [1, 13]. An example is the study by O. Steinbock et al. [33], who demonstrated control of spiral waves in a light-responsive BZ reaction by light-based periodic modulation of excitability. In the present study, optogenetics helps up develop the biological analog of the light-sensitive excitable chemical medium that can be investigated in experimental systems. This feature complements the use of in silico methods to explore fundamental concepts such as possible mechanisms of unsuccessful optical defibrillation during periodic stimulation.

We observed during sub-threshold global periodic illumination with a fixed frequency that a stationary spiral wave starts to meander in complex patterns, and the meandering spiral wave can not be terminated unless it is close enough to a non-excitable boundary or another spiral core of opposite topological charge. Based on this observation, we investigated a more tailored approach for controlling such spirals, which involved adjusting the stimulation frequency to the frequency of the illuminated spiral wave using feedback from a measuring electrode. This allows us to exert subtle control over the spiral wave dynamics, enabling us to steer the wave in a desired direction, which is determined by the position of the measuring electrode.

**Fig. 4:**
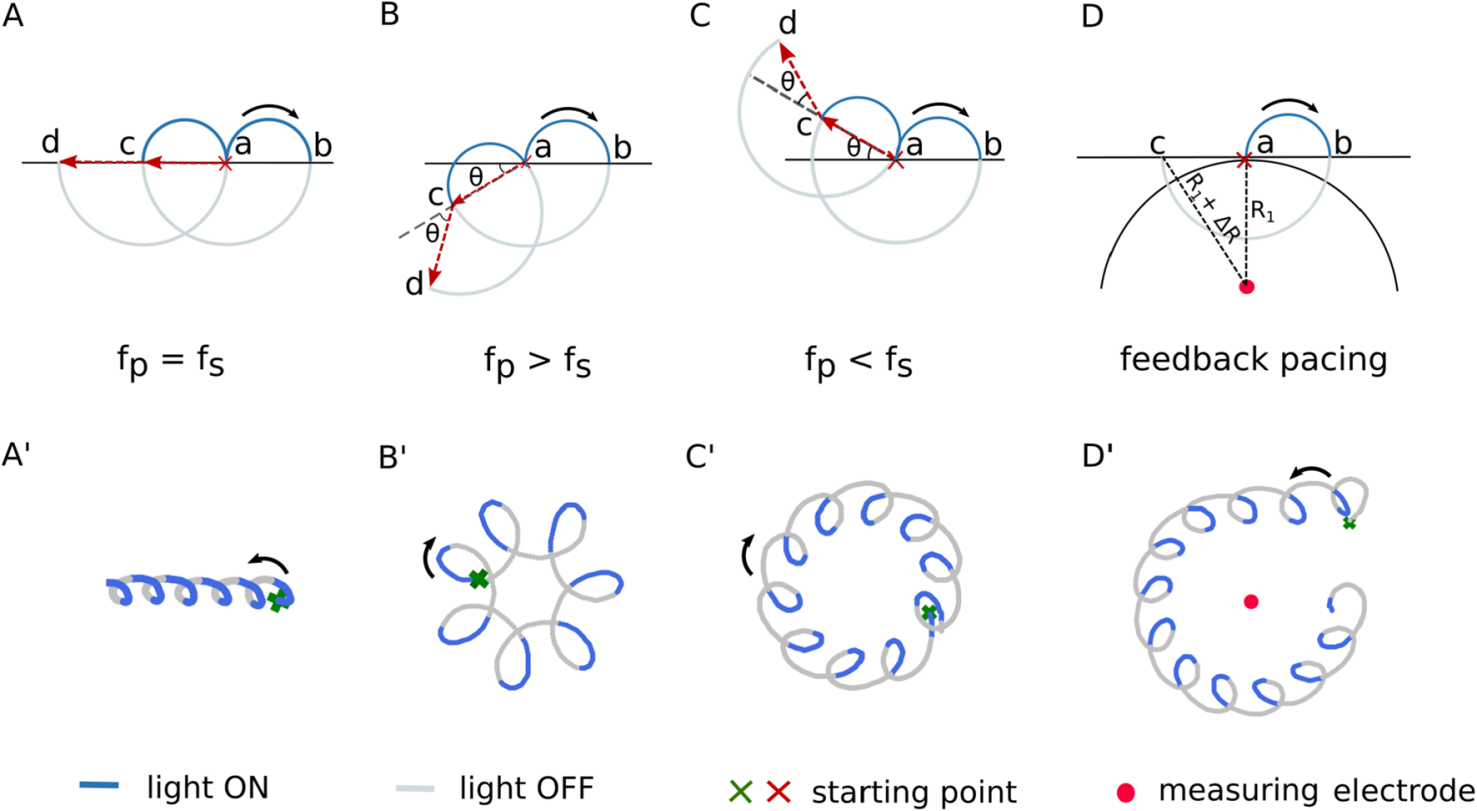
Spiral wave’s core trajectory during open-loop and closed-loop optical pacing. **A** Schematic sketch of the trajectory of the spiral wave’s core during resonant drift with pacing frequency (*f*_p_) equal to the rotation frequency of the spiral wave (*f*_s_). Tip of the spiral wave drifts from “a” to “d” after two rotations with two optical pulses (blue). **A'** Trajectory of the spiral wave tip during six rotations in a numerical simulation. **B** Schematic representation of hypocycloidal meander at *f*_p_ > *f*_s_. The tip of the spiral wave moves from “a” to “d” displaced at an angle of 2*θ* with respect to the horizontal. **B'** Trajectory of the spiral wave tip during five rotations in a numerical simulation. **C** Schematic representation of epicycloidal meander at *f*_p_ < *f*_s_. The spiral tip moves from “a” to “d” displacing at an angle of 2*θ* with respect to the horizontal. **C’** Trajectory of a spiral wave tip during ten rotations in a numerical simulation. **D** Schematic representation of the spiral wave core during resonant feedback pacing. After one full rotation, the tip travels from a to c with an increased distance (∆R) respect to measuring electrode shown in red dot. **D'** Trajectory of the spiral wave tip during 12 rotations in a numerical simulation

To better understand how the trajectory of a spiral wave tip evolves in heart tissue whose excitability is temporally modulated using a sequence of sub-threshold optical pulses, we present a schematic representation of the modulated core dynamics. Note that in the complete absence of modulation, the tip of the spiral wave traces a circular core in the given simulation domain (see Fig. 1A). When light is applied, the radius of this circle decreases as the core size is reduced. Consequently, the curvature of the tip trajectory increases. However, removing the light restores the original core size and the corresponding curvature of the tip trajectory. In the presence of pulsed modulation (open-loop or closed-loop), the curvature of the spiral tip is forced to oscillate between two preset values (one characteristic of the cardiac tissue, and the other determined by the intensity of the applied light stimulation). Figure 4A–D describes the impact of these oscillations on the shape of the tip trajectory, which is marked in blue in the presence of light, and in gray in the absence of it. Figure 4A illustrates the case of open-loop optical pacing with *f*_p_ = *f*_s_; Fig. 4B shows the case of *f*_p_ > *f*_s_; Fig. 4C) demonstrates *f*_p_ < *f*_s_, and Fig. 4D represents the case of closed-loop optical pacing with resonant feedback. In each case, the tip of the spiral wave is initially located at “a”, which is indicated by a red cross. When the light is turned on, the spiral tip moves from “a” to “b” during the first half period of rotation along a path of increased curvature. At “b”, the light goes off. The original curvature of the tip trajectory is restored. Thus for the remaining half period, the spiral rotates in a circle with a larger core radius. The tip then reaches “c”, from where the pattern repeats. When *f*_p_ = *f*_s_, the tip exhibits an effectively linear displacement. Thus, the spiral wave exhibits a resonant drift. Figure 4A' shows an example of the core dynamics of a spiral wave during resonance drift in a simulation study with *f*_p_ = *f*_s_ = 13.33 Hz. When *f*_p_ > *f*_s_, (Fig. 4B) the spiral tip moves from “a” to “b”, whereupon the light is turned off. The core radius is restored and the curvature of the tip trajectory decreases. The spiral tip traces a large sector as in the previous case with *f*_p_ = *f*_s_. However, it is unable to complete half a period of rotation in the absence of light because *f*_p_ is faster than *f*_s_, which results in the early application of the subsequent optical pulse. The net displacement of the spiral tip is at an angle > 180^°^ relative to the displacement in the previous rotation. This pattern is repeated for every modulation cycle and leads to the development of a hypocycloidal meander pattern with the petals pointing outward, (Fig. 4B'). On the other hand, when *f*_p_ < *f*_s_, (Fig. 4C) the spiral tip spends longer time in the absence of light than in the presence of it. Thus, the gray trajectory extends beyond a half period of rotation before the next light pulse is applied. The effective displacement of the spiral tip is at an angle < 180^◦^ relative to the previous cycle of modulation. This leads to the development of an epicycloidal meander pattern with inward-pointing petals, as shown in Fig. 4C'. The core dynamics during resonant feedback are shown in Fig. 4D. In this case, the spiral wave tip is initially located at a distance R1 from the measuring electrode (red circle). When the first light pulse is applied, the curvature of the tip trajectory increases as the tip moves from “a” to “b”. Subsequently, when the light is turned off, the original curvature is restored and the spiral tip moves from “b” to “c” along the gray trajectory. But, as the distance of the tip from the measuring electrode consequently increases from R1 to R1 + ∆R, the second optical pulse is applied with a delay time causing the spiral wave to be underdriven with respect to the measuring electrode. Thus a meander pattern is generated with inward pointing petals encircling the measuring electrode. Figure 4D' shows a simulation of the core dynamics during resonant feedback of a spiral wave.

It is important to mention that in this work we used only a single light intensity and pulse length to gain mechanistic insight into the dynamics of the spiral wave in successful and unsuccessful termination with global homogeneous illumination. However, light intensity and pulse width do indeed have an influence over the electrical behavior at the cell level which eventually influences propagation in cardiac tissue. In fact, the width of the applied pulse has a direct bearing on the direction of motion of the spiral tip, whereas light intensity has an effect on the velocity of the propagating excitation. Shorter or longer pulse widths can change the number of petals in complex meander patterns, whereas linearly drifting spirals can be forced to change direction so that their effective paths deviate from linearity. Application of higher sub-threshold light intensities can, on the other hand, speed up a drifting spiral until the supra-threshold regime is reached. Similarly, the homogeneity of distribution of the light-gated ion channels is an important factor when it comes to real cardiac tissue, as this impacts both the direction and velocity of drift. With a non-homogeneous expression of the light-gated ion channel, feedback resonance should still be observed, but with interruptions in the meandering trajectory, which can delay the process of termination. In cases where the non-homogeneity of illumination is substantial or where the spatial distribution/expression of channelrhodopsin locally interrupts a feedback loop, we can also expect the failure of the termination process.

In a previous study [20], we investigated the control of a single spiral wave using spatial modulation of cardiac excitability. For this purpose, we used different structured and global sub-threshold illumination patterns in an optogenetically modified 2D model of mouse ventricular tissue. Our studies have shown that it is possible to cause a spiral wave drift by spatial modulation of cardiac excitability. This drift was observed along the illumination gradient, a possible scenario that may occur during global surface illumination of the heart, in which an illumination gradient pattern forms below the surface because of the transmural decay of light. This allowed us to gain better insight into the mechanisms of arrhythmia termination during global optical defibrillation. In addition, Li et al. [23] investigated the control of the dynamics of a spiral wave in a 2D cardiac tissue model via the temporal modulation of cardiac excitability. They reported the resonant drift dynamics of this spiral wave when the stimulation frequency is close to the frequency of the spiral wave. However, in the current study, we show that periodic stimulation (with a frequency close to the spiral waves) does not necessarily lead to a resonant drift because the frequency of the spiral wave alters (depending on light intensity) during illumination. This leads to different meander patterns, which could be the reason for the unsuccessful termination. Depending on the pacing frequency we observed different core dynamics with meandering patterns and resonant drift. If we consider this 2D region as a monolayer of the heart during global surface illumination, we get a better mechanistic insight into how the spiral waves that rotate beneath the surface of the heart and experience sub-threshold intensity can be successfully or unsuccessfully controlled.

In the studies of Biktashev et al. [5, 7] a deviation of resonant drift from linearity due to inhomogeneities in the heart tissue was observed. Biktashev et al*.* demonstrated the possibility of repulsion and continuation of the resonance drift away from unexcitable domain boundaries. However, such deviations can be overcome in a robust manner by applying stimulation with resonant feedback [18, 31, 37]. A measuring electrode is used to continuously update the frequency of the applied stimulation based on the change in the rotational frequency of the spiral wave. In this study, although the spiral wave rotates in a homogeneous region, its frequency is affected by illumination. Resonant feedback pacing is a reliable approach to control and steer the spiral wave in any desired direction by placing the electrode in a suitable position.

In vitro experiments are another way to study the dynamics of spiral waves [10, 15, 25]. This provides a better understanding of how efficiently these nonlinear dynamical waves can be controlled in an intact heart. In feedback-controlled resonant pacing, the measuring electrode is placed on the surface of the heart. A limitation is that only the electrical activity of the near-surface spiral waves can be recorded with this electrode. Thus, when the near-surface spiral waves are terminated by sub-threshold illumination, feedback triggering is stopped because no electrical activity is recorded by the measurement electrode. This may cause the spiral waves further below the heart surface to remain unaffected. However, this scenario could be different if the heart is paced with supra-threshold illumination. The near-surface spiral waves are terminated by a different mechanism in which the excitation gap is closed, resulting in the termination of the spiral wave. Furthermore, depending on whether the last detected excitation is due to the spiral wave or induced by the applied optical pulse, further pacing is interrupted or continued. In the latter case, the excitation just beneath the surface of the heart would continue to receive optical stimulation, but at a fixed frequency and in an open-loop.

Alternatively, these sub-surface spiral waves may drift under the influence of an illumination gradient created by the transmural exponential decay of light. This drift can lead to the collision of the spiral with another spiral wave or an unexcitable boundary, resulting in annihilation. Finally, the conclusions presented in this paper are derived from a study of a 2D system. Such a system provides a relatively simple and fundamental understanding of the dynamics of cardiac arrhythmias. However, the real heart is 3D with a complex anatomical structure. Therefore, a major limitation of our study is that it does not take into account the geometrical challenges to be faced during the implementation and functioning of the method. One of these challenges is to find an unexcitable boundary close to which the measuring electrode can be placed.

In the real heart, the only unexcitable region is the atrioventricular border. Thus, for the resonant feedback pacing method to work, a good location to place the measurement electrode would be close to this border. Global surface illumination of a heart results in an exponential decay of the light below the surface. This decay can vary transmissively from supra-threshold to sub-threshold regions depending on the light intensity. Thus, different termination mechanisms may be involved in the overall termination of the VT from different layers of the cardiac wall. In the present study and the study by S. Hussaini et al. [20], we show that spatial or temporal modulation of cardiac tissue excitability leads to the drift-induced termination of spiral waves. The 2D domain in our simulations is a simplified representation of a monolayer of cardiac tissue embedded within the cardiac wall. Despite the aforementioned limitations, it is definitely worth trying to implement the feedback-controlled resonant pacing protocol to defibrillate intact mouse hearts in experiments, because they can potentially reveal the existence of sweet spots (if any) in terms of stimulation amplitude and location of the measuring electrode, that would effectuate successful defibrillation with enhanced termination efficiency.

### Supplementary Information

Below is the link to the electronic supplementary material.

Supplementary file1 (DOCX 204 KB)

## Data Availability

Data and materials will be made available upon reasonable request to the corresponding authors, Sayedeh Hussaini (sayedeh.hussaini@ds.mpg.de) and Stefan Luther (stefan.luther@ds.mpg.de).
