# Supplementary figures and images for: In silico optical modulation of spiral wave trajectories in cardiac tissue
Source: Pflugers Arch. 2023 Dec 14;475(12):1453–61. doi: 10.1007/s00424-023-02889-7 (PMC10730633; doi:10.1007/s00424-023-02889-7)

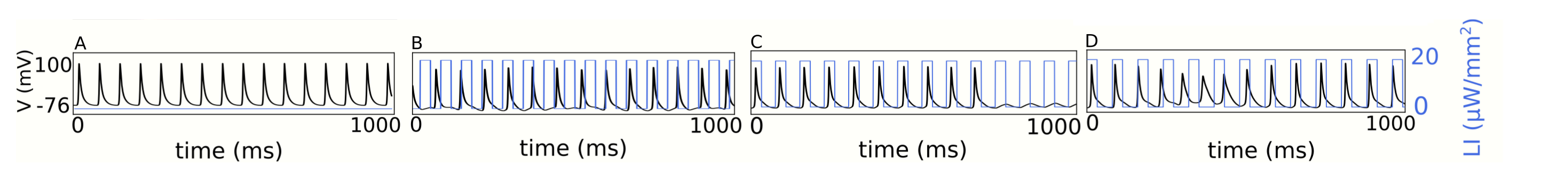

Supplement: Supplementary file 1 — Supplementary file1 (DOCX 204 KB) [file 424_2023_2889_MOESM1_ESM.png]
